# Delayed-onset hypovolemic shock after the Nuss procedure for pectus excavatum

**DOI:** 10.1186/1749-8090-9-15

**Published:** 2014-01-14

**Authors:** Jin Yong Jeong, Jong Hui Suh, Jeong Seob Yoon, Chan Beom Park

**Affiliations:** 1 Department of Thoracic and Cardiovascular Surgery, Incheon St. Mary’s Hospital, The Catholic University of Korea, Incheon, Republic of Korea; 2 Department of Thoracic and Cardiovascular Surgery, Incheon St. Mary’s Hospital, 56, Dongsu-Ro, Bupyeong-Gu, Incheon 403-720, Republic of Korea

**Keywords:** Pectus excavatum, Nuss procedure, Cardiac complication

## Abstract

The Nuss procedure, which is a minimally invasive approach for treating pectus excavatum, has better functional and cosmetic outcomes than other invasive procedures. Cardiac perforation is the most serious complication and several methods for the prevention of intraoperative events has been developed. Although most cardiac injuries are detected in the operating room, in the case described herein the patient experienced sudden hypovolemic shock during the postoperative recovery period. This indicates that special caution is mandatory even after successful execution of the Nuss procedure.

## Background

Since the introduction of minimally invasive repair of pectus excavatum, the Nuss procedure has become the preferred method due to its simplicity, cosmetic effects, and long-term success rate. Complications related to the Nuss procedure are not unusual, but major complications occur rarely. Although cardiac complications are the most disastrous, most cardiac injuries present intraoperatively [1,2] and immediate management is possible. We report herein a rare case of delayed development of hypovolemic shock after a successful Nuss procedure.

## Case presentation

**Figure 1:**
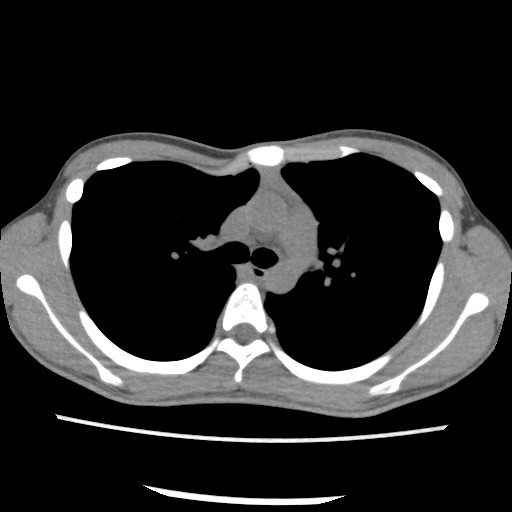
Chest computed tomographic scan showing asymmetric depression of chest wall.

A 17-year-old male presented with exertional dyspnea with a Haller index of 3.46; preoperative chest computed tomography did not reveal any other abnormalities (Figure 1). The patient was placed under endotracheal anesthesia in a supine position. A stainless-steel sternal wire was passed into the deeper portion of the sternal body. The two ends of this wire were attached to a crane device, which was then used to elevate the depressed sternum as described previously [3]. A thoracoscope was introduced into the right pleural space and the pectus clamp was used to begin the dissection of the anterior mediastinum in a cranial position, sliding slowly behind the sternum to reach the optimal level for correction of the pectus excavatum. A chest tube with smooth end-points was then passed through the tunnel created by the pectus clamp so as to form a protective space for the Nuss bar. The bar was passed along the chest tube. The Nuss bar was rotated so that the convexity faced anteriorly to correct the deformity, and then fixed to the chest wall with fixaters. A second Nuss bar was similarly inserted and rotated. The patient tolerated the surgery well, and a postoperative chest x-ray did not reveal any other complications such as pneumothorax or hemothorax.

**Figure 2:**
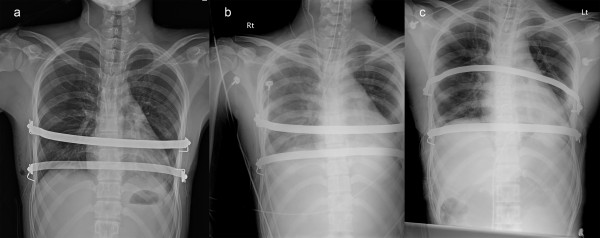
**Serial chest x-ray after operation. (a)** Chest x-ray at the next morning after Nuss procedure showing two bars and hemopneumothorax was not noted **(b)** 2 hours after rounding, right-side hemothorax was revealed. **(c)** After reoperation, hemothorax disappeared.

On the morning after the operation, the patient’s vital signs were stable and there was no evidence of bleeding. A chest x-ray obtained at the morning revealed neither hemothorax nor pneumothorax (Figure 2a). Two hours after the surgeon’s morning rounds, the patient suddenly collapsed and suffered diaphoresis and hypotension. A follow up chest x-ray revealed a right-side hemothorax (Figure 2b). He was transferred immediately to the operating room and after induction of endotracheal anesthesia the right pleural space was examined with a thoracoscope. There was no bleeding focus from the pleural space including the bar insertion site, but there was bleeding from the opening of the anterior pericardium under the lower sternum. The Nuss bars were removed and a lower sternotomy was performed. The bleeding was found to originate from a small laceration of the right ventricle, which was repaired with 4–0 polypropylene sutures. The Nuss bars were reinserted and the patient sent to an intensive care unit. His subsequent postoperative course was uneventful and he was followed without further complications for 1 year.

Cardiac injuries after the Nuss procedure are extremely rare, but surgeons should always be aware of such life-threatening conditions. According to Dr. Nuss’ 20-year experience with minimally invasive surgical repair [4], pneumothorax was the most common complications; cardiac injury was not reported. A recent prospective study found minor complications in 73% of cases, the most common minor complications were breakage of the wires used to secure the stabilizer and bar [1]. Minor pericardial tears developed in seven patients (4.2%) and ten cases of hemopneumothorax were documented during follow-up. Seven cases (4.2%) with major complications were reported, of which three involved significant bar displacement requiring surgical correction. In another case, intraoperative heart injury resulting in perforations in the right atrium and right ventricle had occurred, which were repaired via emergent transverse thoracotomy.

While bar flipping is the most frequent major complication of the Nuss procedure, cardiac injury is always a risk [1,2,5,6] and can lead to life-threatening conditions. Most fatal cardiac injuries arise during passage of the pectus clamp from the right to the left side. The severity of the chest wall deformity, previous surgical correction of pectus excavatum, and history of cardiac surgery creating mediastinal or pleural adhesions are risk factors for cardiac injury.

Several methods for preventing cardiac injury have been proposed. While the use of thoracoscopy is recommended, cardiac perforations have been reported [2]. Some surgeons make a subxiphoid incision for manual guidance of the pectus dissector. The sternal lifting system was introduced to eliminate the risk of cardiac perforation [7] and we used the similar crane for elevation of deeper portion of sternum in the present case [3]. Extrapleural placement of the Nuss bar was suggested and Castellani et al. [1] suggested increasing the anterior convexity of the introducer for deep forms of pectus excavatum. However, in spite of these strategies, there is always a risk of cardiac injury, and meticulous dissection and special attention during both surgery and the postoperative period are necessary to avoid any disastrous consequences.

The reason for the right ventricular injury that occurred in the present case is not clear. As reported elsewhere [1], it may have been caused by passage of the pectus clamp. However, blunt injury by clamp would have been noted in the operating room, but the patient was stable during the surgery. Although it has not been possible to confirm, it may have been caused by the deep sternal wiring used to elevate the sternum with crane. Furthermore, small injuries caused by the sternal wire might have been aggravated by postoperative pain.

While most of the cardiac injuries associated with the Nuss procedure present intraoperatively, some cases of late presentation have been reported [5]. In the present case, the patient underwent successful implantation of Nuss bars with the aid of thoracoscopy and sternal elevation with crane to prevent cardiac injury. The patient was stable during the surgery and his immediate postoperative course was uneventful. However, the patient suddenly collapsed the following morning and further immediate emergent surgery was needed for his recovery.

## Conclusion

This case shows that surgeons should keep in mind the possibility of cardiac injuries during both surgery and the postoperative period. Special caution should be taken even after successful Nuss bar implantation.

## Consent

Written informed consent was obtained from the patient for publication of this Case report and any accompanying images. A copy of the written consent is available for review by the Editor-in-Chief of this journal.

## Competing interest

The authors declare that they have no competing interests.

## Authors’ contribution

Jin Yong Jeong: Drafting the manuscript, Jong Hui Suh: Concept and design of study, Jeong Seob Yoon: Concept and design of study, Chan Beom Park: Critical revision and final approval, All authors read and approved the final manuscript.
